# Potential mechanisms of traditional Chinese medicine in the treatment of liver cirrhosis: a focus on gut microbiota

**DOI:** 10.3389/fmicb.2024.1407991

**Published:** 2024-08-21

**Authors:** Siyuan Sun, Guangheng Zhang, Shimeng Lv, Jinhui Sun

**Affiliations:** ^1^First Clinical Medical College, Beijing University of Chinese Medicine, Beijing, China; ^2^First Clinical Medical College, Shandong University of Traditional Chinese Medicine, Jinan, China; ^3^Gastroenterology Department, Dongzhimen Hospital, Beijing University of Chinese Medicine, Beijing, China

**Keywords:** liver cirrhosis, gut microbiota, traditional Chinese medicine, natural products, microbial metabolites

## Abstract

Cirrhosis, a pathological stage that develops from various chronic liver diseases, is characterized by liver fibrosis, pseudolobular formation, and chronic inflammation. When it progresses to the decompensated phase, the mortality rate of cirrhosis can reach 80%. The role of gut microbiota in the progression of liver diseases has received significant attention. Numerous studies have shown that regulating gut microbiota has significant therapeutic effects on preventing and reversing liver cirrhosis. This article reviewed the mechanisms by which gut microbiota influence liver cirrhosis, explaining the effective therapeutic effects of traditional Chinese medicine. Through multi-directional regulation involving signaling pathways, gut microbiota diversity, and restoration of intestinal barrier function, traditional Chinese medicine has been promising in ameliorating liver cirrhosis, providing treatment options and pharmacological guidance for the occurrence and development of liver cirrhosis.

## 1 Introduction

More than 2 million people worldwide die from liver disease annually, accounting for 4% of all deaths, mainly due to liver cirrhosis and liver cancer (Devarbhavi et al., 2023). As the disease progresses, various complications of liver cirrhosis are key prognostic factors. Data show that about 4–12% of patients with cirrhosis experience more than one decompensated event per year [ascites, variceal bleeding, hepatic encephalopathy (HE)], and progression of cirrhosis to HE occurs in more than 40% of patients over a 5-year period. Secondly, during hospitalization, 25–50% of patients with cirrhosis develop bacterial infections, and 20% of patients suffer from acute kidney injury (Moon et al., 2020). The above data are more conservative for European countries (Peng et al., 2016; Pimpin et al., 2018). Given that liver cirrhosis and its complications are still a major burden in the global health problem, timely reverse this is crucial.

The etiology of liver cirrhosis is complex and multifaceted, with common causes involving viral hepatitis, alcohol-related liver disease (ALD), and non-alcoholic fatty liver disease (NAFLD). Epidemiological data reveal that over 40% of global liver cirrhosis cases are infected with hepatitis B virus, while more than 20% are infected with hepatitis C virus (Huang et al., 2023). In addition to alcohol consumption, factors such as obesity, type 2 diabetes, metabolic syndrome, and hypertension also increase the likelihood of chronic liver disease progressing to cirrhosis (Younossi, 2019). These factors gradually lead to liver cell damage, degeneration and necrosis, and then liver cell regeneration and fibrous connective tissue proliferation, liver fibrosis formation, and finally progress to cirrhosis. Its pathological evolution is mainly composed of the following four aspects: (1) The role of pathogenic factors leads to extensive degeneration and necrosis of hepatocytes and collapse of the reticular fiber scaffolds of hepatic lobules (Romanelli and Stasi, 2016); (2) The remaining hepatocytes are not regenerated along the original scaffold, but form irregular nodular clusters of hepatocytes (regenerated nodules) (Iredale, 2007). (3) Various cytokines (such as TGF-β1) promote the production of fibrosis, extending and expanding in the central venous area and the portal area of the lobule, forming fibrous septa (Nishimura et al., 2021); (4) The proliferative fiber tissue makes these fiber intervals interconnect, wrap around the regenerated nodules or re-segment the residual hepatic lobules and transform them into false lobules, forming the typical morphological changes of cirrhosis. The above pathological changes caused the narrowing, occlusion and distortion of the vascular bed, The vessels were squeezed by regenerating nodules, and the branches of intrahepatic portal vein, hepatic vein and hepatic artery lost their normal relationship, and anastomosed branches appeared (Pu et al., 2023). Hepatic blood circulation disorder is the pathological basis of portal hypertension, aggravates hepatic cell ischemia and hypoxia, and promotes the further development of cirrhosis (Kaji and Yoshiji, 2020; Mahmoudi et al., 2022; Violi et al., 2023). Clinically, liver cirrhosis is roughly divided into compensated and decompensated liver function. Most patients in the compensatory stage are asymptomatic or have mild symptoms, and there are no abnormalities or mild abnormalities in liver function tests; when the stage progresses to decompensation, patients will have significant symptoms, mainly manifested by liver function decline and portal hypertension. The transition from compensated to decompensated cirrhosis represents a significant reduction in life expectancy and an increase in mortality (Ginès et al., 2021). Therefore, it is of great significance to accurately grasp the “critical point” of progression from compensated to decompensated and delay or reverse the progression of early liver cirrhosis to improve the quality of life and survival rate of patients.

In recent years, numerous studies have highlighted the close relationship between gut health and the normal functioning of various physiological systems, particularly the dynamic balance between the gut and the liver via the portal vein system. With the progression to the decompensated stage, the gut microbiota changes progressively in the direction of more serious disorders, and conversely, the gut microbiota dysbiosis will also break the balance state of enterohepatic circulation, affect the occurrence and development of liver diseases, and become an important driving factor for complications such as hepatic encephalopathy (HE) and spontaneous bacterial peritonitis (SBP) (Trebicka et al., 2021). In other words, there is a causal relationship between the gut microbiota and cirrhosis. Therefore, early regulation of gut microbiota is expected to become a new approach for liver cirrhosis treatment (Tsiaoussis et al., 2015). Compared to Western medicine, traditional Chinese medicine (TCM) is more widely accepted by patients owing to its advantages, including fewer adverse effects, enhanced specificity, reduced dependency, and diminished drug resistance. TCM has been widely used in the treatment of various clinical diseases. For example, flavonoids directly regulate insulin secretion, glucose metabolism-related enzymes, and related signaling pathways, thereby playing a significant role in treating diabetes and its complications (Bai et al., 2019). Similarly, natural products also have significant implications in regulating intestinal microecology and treating liver cirrhosis. There is a two-way promoting effect between citrus fruits rich in flavanone and intestinal flora. Intestinal flora plays a significant metabolic potential for polyphenols. Polyphenols can also inversely increase the abundance of beneficial bacteria, such as *Bifidobacterium* and *Lactobacillus* spp., and positively regulate the production of SCFA and intestinal PH value. Thus, the bacteria and the host can achieve a win-win situation (Tang et al., 2022). Baicalin mitigates diethylnitrosamine-induced cirrhosis by inhibiting oxidative stress and inflammation (Wang et al., 2024). Forsythiside A (FTA) demonstrates its good hepatoprotective effects by improving liver fibrosis by reversing the decreased bacterial richness, displaying anti-inflammatory and antioxidant properties, and improving the metabolism of bile acids (BAs) (Zhao et al., 2021). Emodin, belonging to the anthraquinone class of compounds, has hepatoprotective and anticancer effects against liver damage induced by various factors such as alcohol, high-fat diet (HFD), and lipopolysaccharides (LPS) (Hu et al., 2020).

**Figure 1:**
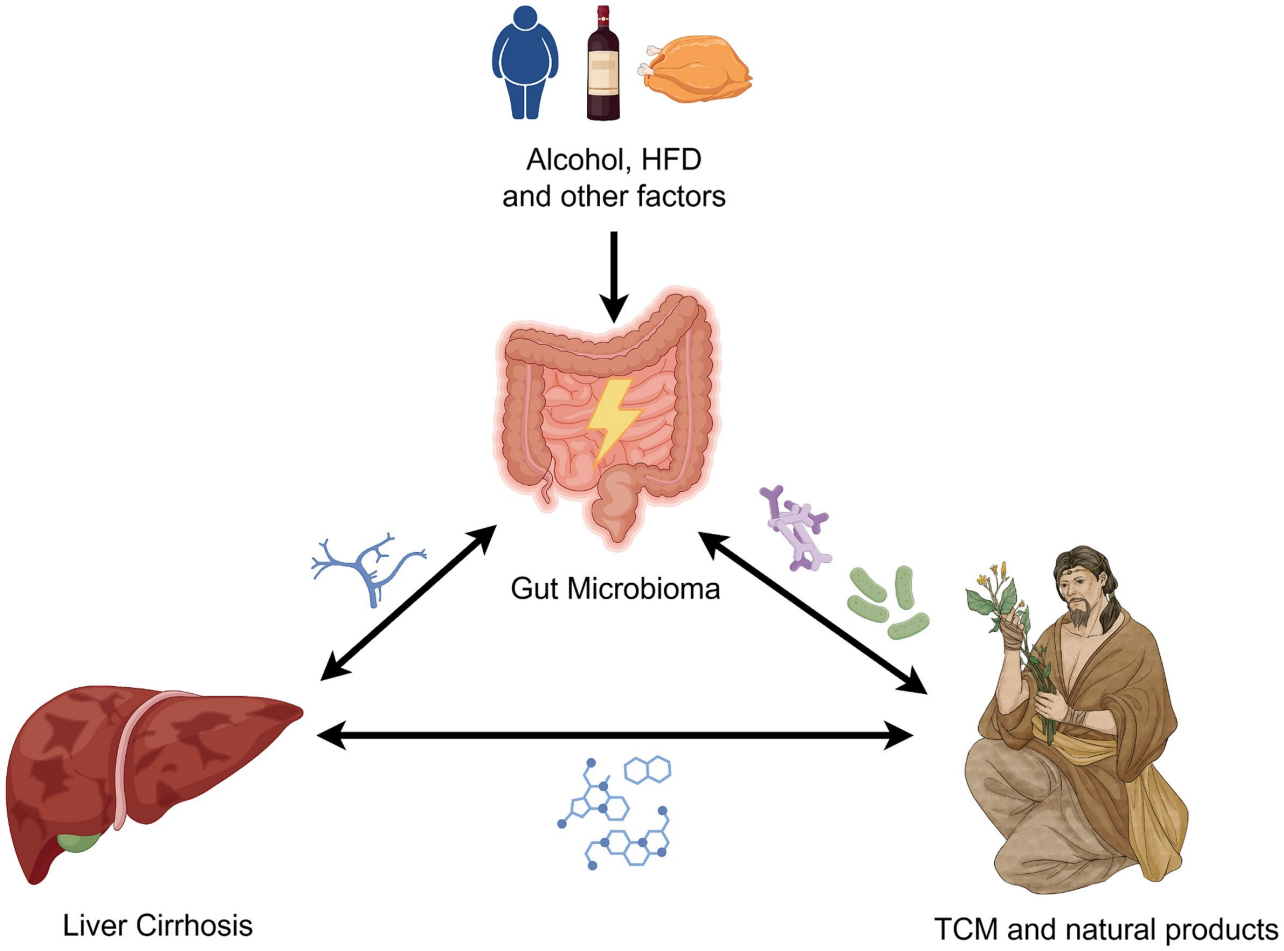
Links among liver cirrhosis, gut microbiota, and TCM. The figure is drawn with Figdraw.com.

Based on the close relationship between the intestinal flora and the liver, the aim of this study was to explore the role and potential mechanisms of gut ecological imbalance in liver cirrhosis. Furthermore, the study also aimed to assess the feasibility of TCM interventions targeting gut microbiota to improve liver cirrhosis, providing insights for future research directions and clinical management strategies. The interplay among liver cirrhosis, gut microbiota, and TCM is illustrated in Figure 1.

## 2 Gut microbiota and cirrhosis

The human body is consisted by a complex ecosystem, including bacteria, fungi, viruses, and archaea, which typically coexist with the host to maintain homeostasis. The microbial community is extensive and diverse. The number of bacterial cells in humans is tenfold higher than that of animals, with over 70% of bacteria concentrated within the intestine (Neish, 2009). The gut microbiota primarily consists of anaerobic bacteria (Lynch and Pedersen, 2016), with Firmicutes and Bacteroidetes as dominant species. Studies have indicated that changes in the ratio of Firmicutes to Bacteroidetes might affect lipid metabolism and the central nervous system, potentially leading to obesity (Ley et al., 2005; Turnbaugh et al., 2006; Koliada et al., 2017). In recent years, an increasing number of researchers have analyzed the close relationship between gut microbiota and human physiology and pathology from various perspectives, such as the brain-gut axis, liver-gut axis, and lung-gut axis. Shao et al. demonstrated a significant alleviating effect on depression by targeting the brain-gut axis to regulate the peripheral microenvironment (Shao et al., 2023). Li et al. (2023) found that the lung-gut axis plays an important role in various pulmonary diseases such as COVID-19 and asthma, by reshaping the gut microbiota in rat models.

Therefore, the balance of gut microbiota is crucial for human health and diseases, often referred to as the “new virtual metabolic organ” within the body (Lankelma et al., 2017). At birth, a baby’s gut microbiota begins to establish itself. Disruptions in the composition and diversity of intestinal bacteria during early childhood, termed ecological imbalance, significantly impacts metabolism and immunity in adulthood (Milani et al., 2017). The gut and liver interact through various pathways, including the biliary tract and systemic circulation and the enterohepatic circulation facilitated by the portal vein (Giuffrè et al., 2020). Nutrients and microbial metabolites absorbed in the intestine enter the liver via the portal vein, where they are absorbed or stored in hepatic cells. Microorganisms and their metabolites are closely related to the immune and metabolic functions of the liver (Hsu and Schnabl, 2023). Conversely, the liver uses various immune cells, such as Kupffer cells, NKT cells, and Th17 cells, to regulate the intestinal barrier, prevent bacterial translocation (BT), and inhibit inflammatory responses (Gola et al., 2021). Besides, the maturation of gut microbiota is closely related to the synthesis and transport of BAs. Oral administration of BAs to postnatal mice can increase the richness and maturity of their gut microbiota (Van Best et al., 2020). Similarly, Lv et al. (2016) using metagenomic sequencing, observed a decrease in beneficial gut bacteria and an increase in Firmicutes and Proteobacteria phyla, considered opportunistic pathogens, in cholestatic patients. Additionally, upon conversion into secondary BAs, signals are transmitted through the nuclear farnesoid X receptor (FXR) and G-protein-coupled membrane receptor 5 (TGR5) in the intestinal epithelium to regulate host metabolism (Wahlström et al., 2016). In summary, the connection between gut microbiota and the liver is bidirectional, sustaining host health through synergistic effects.

The gut microbiota is widely involved in host metabolism, and its dysbiosis can alter the host’s metabolic phenotype. Intestinal ecological disorders are closely related to cirrhosis and end-stage liver disease. Li et al. conducted a quantitative metagenomic association analysis on 98 patients with liver cirrhosis and 83 healthy controls, revealing a significant difference in the abundance of 75,245 genes believed to be related to liver cirrhosis (Qin et al., 2014). Similarly, an analysis of the fecal microbiome of patients with cirrhosis resulting from alcohol consumption and hepatitis B virus infection demonstrated a decrease in functional genes related to amino acids, lipids, nucleotides, and other metabolic pathways. The abundance of these functional genes negatively correlated with the Child-Pugh score (Chen et al., 2014), indicating that the structure and function of intestinal flora are significant in cirrhosis and its diverse complications. Therefore, exploring the changes in gut microbiota among patients with liver cirrhosis is expected to aid in intervening in the development of liver cirrhosis and its associated complications by targeting gut microbiota as a therapeutic approach.

## 3 Mechanisms of gut microbiota involved in the occurrence and development of live cirrhosis

**Figure 2:**
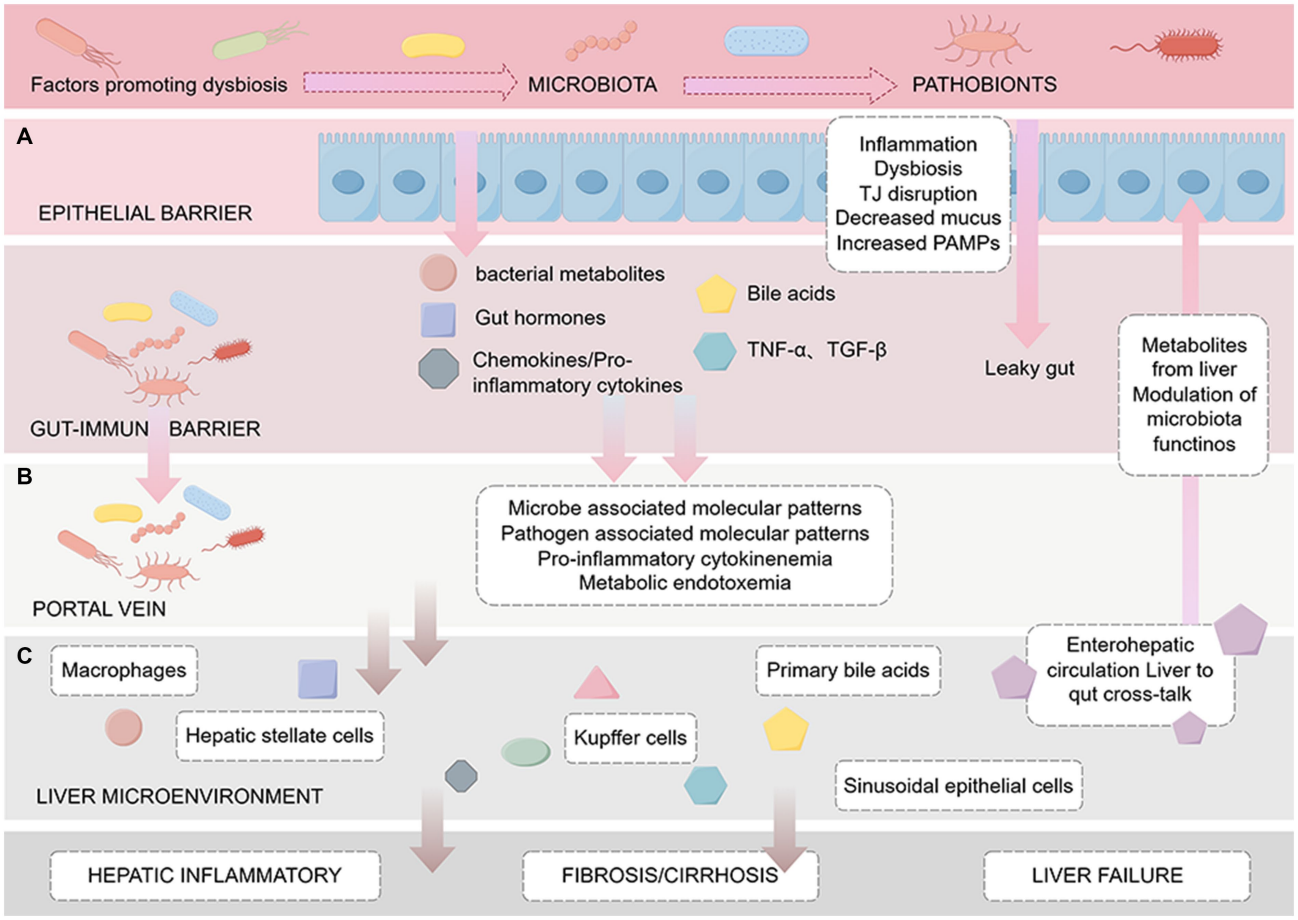
Pathogenesis of cirrhosis based on the enterohepatic circulation. The figure is drawn with Figdraw.com. The figure is divided into three sections from top to bottom: **(A)** Intestinal barrier: The effect of intestinal microbes, metabolites such as BAs and endotoxin on intestinal mucosal barrier function, including increased permeability and bacterial translocation. **(B)** Portal vein: Factors delivered to the liver via the portal vein, such as cytokines and microbiota-associated molecular patterns (MAMPs), which induce the activation of immune cells such as Kupffer cells, leading to inflammation and endotoxemia. **(C)** Liver microenvironment: After microbial-derived metabolites reach the liver through the portal vein, they trigger a series of reactions in the liver, including the activation of macrophages and HSCs, and may lead to liver fibrosis/cirrhosis, portal hypertension, and eventually liver failure.

The occurrence and development of liver cirrhosis are significantly influenced by the gut microbiota. Research indicates that changes in the gut microbiome can damage the liver by inducing inflammatory responses, exacerbating liver fibrosis, and affecting host metabolism (Martínez-Montoro et al., 2022; Wang et al., 2022; Zhang et al., 2023). Moreover, greater changes in the gut microbiota are associated with an increased risk of infection and reduced survival in liver cirrhosis (Fang et al., 2022). The gut microbiota known to be involved in the occurrence and development of chronic liver diseases such as cirrhosis. However, the specific mechanism by which changes in the gut microbiota leads to cirrhosis remains unclear. Current hypotheses propose mechanisms involving ecological dysbiosis, intestinal barrier disruption, abnormal enterohepatic circulation and BA metabolism, and the translocation of microbial-derived metabolites (Figure 2).

### 3.1 Dysbiosis of gut microbiota

Dysbiosis of the gut microbiota mainly involves quantitative and qualitative changes in intestinal flora, where qualitative changes mainly lie in the composition and quantity of bacteria (Wegierska et al., 2022). Compared to the healthy population, the gut microbiota of patients with liver cirrhosis increases in Proteobacteria and Clostridium but decreases in Bacteroidetes at the phylum level (Qin et al., 2014; Bajaj et al., 2015; Trebicka et al., 2021). There is a reduction in the Bacteroides genus, with significant enrichment observed in Veillonella, Streptococcus, Clostridium, and the β-Proteus genus (Qin et al., 2014). Marco et al. demonstrated lower levels of Lachnospiraceae in fecal samples from patients with liver cirrhosis compared to healthy individuals, highlighting its important role in liver immune and metabolic processes through the production of short-chain fatty acids (SCFAs) (Zamparelli et al., 2017). In addition, beneficial bacteria like Ruminococcaceae and Clostridium Cluster XIV showed a significant decrease in the proportion of gut microbiota in patients with liver cirrhosis (Bajaj, 2019). Bajaj et al. introduced the cirrhosis dysbiosis ratio (CDR) to reflect the ratio of native to non-native microbiota in their fecal microbiota, and demonstrated correlation between CDR and the severity of liver cirrhosis, which further confirmed the significance of gut microbiota diversity in disease progression (Bajaj et al., 2014). The significant reduction of Clostridium XIV, Ruminococcae, and Trichrospiridae, which belong to the native flora, and the enrichment of potential pathogens Enterobacteriaceae and Bacteroidetes, synergistically promoted the accumulation of endotoxins in metabolites, and endotoxin levels could be used as an important indicator of the severity of HE. In addition, the abundance of Actinomycetes, Bacteroidetes and Firmicutes decreased to varying degrees in patients with cirrhosis infection, suggesting a causal relationship with the increased risk of systemic inflammation in patients with cirrhosis (Kulkarni et al., 2024). Families such as Lachnospiraceae and Ruminococcus, similar to acetate producers, are abundant in healthy individuals but significantly insufficient in patients with liver cirrhosis. Pathogenic bacterial overgrowth includes Enterobacteriaceae, Staphylococcus, and Enterococcus (Chen et al., 2011). *Veillonella atypica*, *Veillonella parvula*, and the Streptococcus genus were identified as pivotal components in disrupted gut microbiota of patients with liver cirrhosis. These bacteria produce rich sialidase enzymes that degrade human mucin O-glycans, leading to intestinal barrier disruption (Patel et al., 2022). Additionally, these bacteria trigger the secretion of chemokines such as CXCL8 and tumor necrosis factor-α (TNF-α) (Horvath et al., 2019), inducing inflammatory responses and increasing intestinal permeability (Chen et al., 2015). The phenomenon of oral colonization bacteria expanding in the intestine may be attributed to reduced gastric acid secretion, diminishing bacterial clearance in the stomach (Tsuda et al., 2015). Notably, Bifidobacteria and Lactobacillus, typically regarded as beneficial gut bacteria with anti-inflammatory properties (Yu et al., 2021; Song et al., 2023), are enriched in the intestinal tract of patients with alcoholic cirrhosis, suggesting potential adverse effects in liver cirrhosis patients. Therefore, attention should be paid to the administration of medications to such patients (Dubinkina et al., 2017).

Quantitative changes in gut microbiota are mainly characterized by small intestinal bacterial overgrowth (SIBO), which is related to factors such as decreased intestinal peristalsis, reduced gastric acid secretion, and impaired BA secretion, leading to prolonged content retention in the small intestine (Nishimura et al., 2021; Efremova et al., 2023). SIBO has been shown to be a risk factor for clinical decompensation of liver cirrhosis, which is manifested by increased abundance of *Firmicutes* and *Fusobacterium* at the phylum level; at the genus level, the abundance of *Blautia*, *Fusicatenibacter*, *Acinetobacter*, *Oribacterium*, and *Haemophilus* increased, with *Blautia* as a major contributor to the induction of SIBO in all overproliferating bacteria. Cirrhotic patients with SIBO had more severe intestinal flora dysbiosis, characterized by a higher Firmicutes/Bacteroidetes ratio, compared to those without SIBO (Maslennikov et al., 2022), suggesting this ratio as a crucial indicator reflecting cirrhosis progression. SIBO facilitates the translocation of harmful microorganisms and their metabolites (especially LPS) due to increased intestinal permeability, finally leading to endotoxemia (Augustyn et al., 2019). Activation of NF-κβ by endotoxemia triggers the production of pro-inflammatory cytokines, inducing liver inflammation and insulin resistance (IR), a significant factor in the pathogenesis of cirrhosis and its complications (Clarembeau et al., 2020). Moreover, SIBO generates excess acetate and disrupts the homeostasis of BA metabolism, promoting the deconjugation of BA (Bala et al., 2006). Acetate harms the colon mucosa, while unbound BAs adversely affect lipid metabolism and intestinal mucosa, accelerating the progression of liver cirrhosis (Nafday et al., 2005; Hartmann et al., 2018). The diversity of fecal microbiota in patients in the decompensated stage was lower than that in the compensated stage, and the *Clostridium* XIV, Ruminococcaceae and Trichromyceae belonging to the native flora were significantly reduced, while the potential pathogenic bacteria Enterobacteriaceae and Bacteroides were enriched, and these two changes synergistically promoted the accumulation of metabolite endotoxins, and the increase of gut permeability led to SIBO and pathological BT. The overall translocation of viable bacteria to mesenteric lymph nodes is an important feature of decompensated cirrhosis, which in turn can damage the host’s systemic and local immune defenses, leading to further exacerbation and a vicious cycle (Giannelli et al., 2014). A meta-analysis involving 306 samples revealed a significant correlation between SIBO and various complications of liver cirrhosis, such as HE, spontaneous bacterial peritonitis, malnutrition, and ascites (Maslennikov et al., 2018). However, due to the lack of an optimal diagnostic standard for SIBO (Massey and Wald, 2021), it cannot be definitively concluded that SIBO directly leads to these complications, necessitating further research for confirmation. The abundance changes of gut microbiota in patients with liver cirrhosis are shown in Table 1.

**Table 1:** Changes in gut microbiota abundance in patients with cirrhosis.

Classification	Common bacteria species	Change trend	Mechanism	Reference
Phylum	*Bacteroidetes*	Down	The synthesis of SCFAs (acetate, butylhydrochloric acid and palmitate) decreased; Secondary BAs production was insufficient and BA uncoupling was impaired.	Qin et al. (2014), Zamparelli et al. (2017), and Bajaj (2019)
	*Proteobacteria*	Up	Promoting the translocation of cells and bacteria, making them the most harmful bacteria in the gut microbiota, has the potential to stimulate liver fibrosis.	Zamparelli et al. (2017) and Maslennikov et al. (2018)
	*Fusobacteria*	Up	The transformation from primary BA to secondary BA was disordered. It produces LPS that travels with the impaired intestinal barrier into the liver, increases oxidative stress and activates TLR4, producing proinflammatory and profibrotic effects.	Zamparelli et al. (2017)
Family	*Enterobacteriaceae*	Up	It releases potent endotoxins and produces endogenous ethanol, which disrupts intestinal barrier function, increases intestinal permeability, stimulates the innate immune system, and leads to chronic inflammation of the intestinal wall and liver.	Bajaj et al. (2014), Bajaj (2019), and Massey and Wald (2021)
	*Veillonelaceae*	Up	It is the most representative fibrosis-associated bacterium and positively interacts with ursodeoxycholic acid and propionate.	Wahlström et al. (2016) and Kulkarni et al. (2024)
	*Streptococcaceae*	Up	It was negatively correlated with acetic acid level and positively correlated with Child-Pugh score.	Wahlström et al. (2016)
	*Lachnospiraceae*	Down	Reduction of 7α-dehydroxylation; Inhibition of this bacterium leads to a decrease in SCFA production, which in turn raises colonic PH and increases ammonia content.	Maslennikov et al. (2018), Bajaj (2019), and Kulkarni et al. (2024)
	*Ruminococcaceae*	Down	It affects the 7α-dehydroxylation process. The production of SCFA decreased, and the levels of inflammatory cytokines in the blood are increased.	Wahlström et al. (2016), Maslennikov et al. (2018), Bajaj (2019), and Kulkarni et al. (2024)
	*Enterococcaceae*	Up	Promote endotoxin release, cause intestinal barrier damage and BT; It activates proinflammatory cytokines, exacerbates hepatocyte injury, and plays a key role in the complications of infectious cirrhosis, especially SBP.	Kulkarni et al. (2024), Zamparelli et al. (2017), and Maslennikov et al. (2023)
	*Porphyromonadacea*	Up	Promoting inflammation and disrupting the normal microbiome; It is associated with cognitive impairment in patients with liver cirrhosis.	Maslennikov et al. (2023)
	*Alcaligeneceae*	Up	It is associated with bacterial infection in patients with liver cirrhosis.	Maslennikov et al. (2023)
	*Staphylococcaceae*	Up	It can change gastric hemodynamics and increase the incidence and severity of portal hypertensive gastropathy. It is also closely related to liver failure and endotoxemia.	Zamparelli et al. (2017) and Kulkarni et al. (2024)
Genus	*Bifidobacterium*	Up	It was negatively correlated with the expression level of aspartate aminotransferase and prothrombin time.	Yu et al. (2021)
	*Lactobacillus*	Up	Promoting the production and accumulation of endogenous ethanol.	Zamparelli et al. (2017) and Yu et al. (2021)
	*Faecalibacterium prausnitzii*	Down	The production of butyrate is reduced, weakening its anti-inflammatory protective and oxidative stress-ameliorating properties in the gut.	Wahlström et al. (2016)
	*Prevotella*	Up	Promotes ethanol metabolism in the intestine.	Wahlström et al. (2016) and Manzoor et al. (2022)
	*Clostridium*	Down	Involved in 7α-dehydroxylation, the reduction of its genus leads to a lack of substrates for the conversion of primary BA to secondary BA.	Wahlström et al. (2016) and Zamparelli et al. (2017), and Maslennikov et al. (2018)
	*Blautia*	Down	The production of SCFA decreased; BA transformation regulation is limited; It also attenuated its anti-inflammatory properties.	Bajaj et al. (2012) and Meijnikman et al. (2022)	
	*Oscillospira*	Down	Reduced production of SCFA with nutritional, metabolic, and immunomodulatory effects.	Baltazar-Díaz et al. (2022)
	*Pseudobutyrivibrio*	Down		Zamparelli et al. (2017)
	*Coprococcus*	Down		Wahlström et al. (2016) and Bajaj (2019)
	*Roseburia*	Down		Qin et al. (2014) and Zamparelli et al. (2017)
	*Atopobium*	Up	Promotes the production of high levels of CH_3_SH, which directly contributes to the occurrence of hepatic encephalopathy.	Manzoor et al. (2022)
	*Veillonella*	Up	It produces propionate, which has proinflammatory features.	Chen et al. (2011), Chen et al. (2016), and Wahlström et al. (2016)
	*Dialister*	Up		Chen et al. (2016), Manzoor et al. (2022), and Wu et al., 2023
	*Megasphaera*	Up		Chen et al., 2016 and Manzoor et al. (2022)
	*Akkermansia muciniphila*	Down	It reduces the production of intestinal mucus and TJ expression, weakens the protective function of the intestinal barrier, and aggravates liver injury and disease susceptibility.	Lee et al. (2020) and Liu et al. (2019)
	*Faecalibacterium prausnitzii*	Down	As a key beneficial commensal, its reduction in abundance attenuated the anti-inflammatory effect on the liver.	Ponziani et al. (2018) and Oh et al (2020)

### 3.2 Intestinal barrier dysfunction

**Figure 3:**
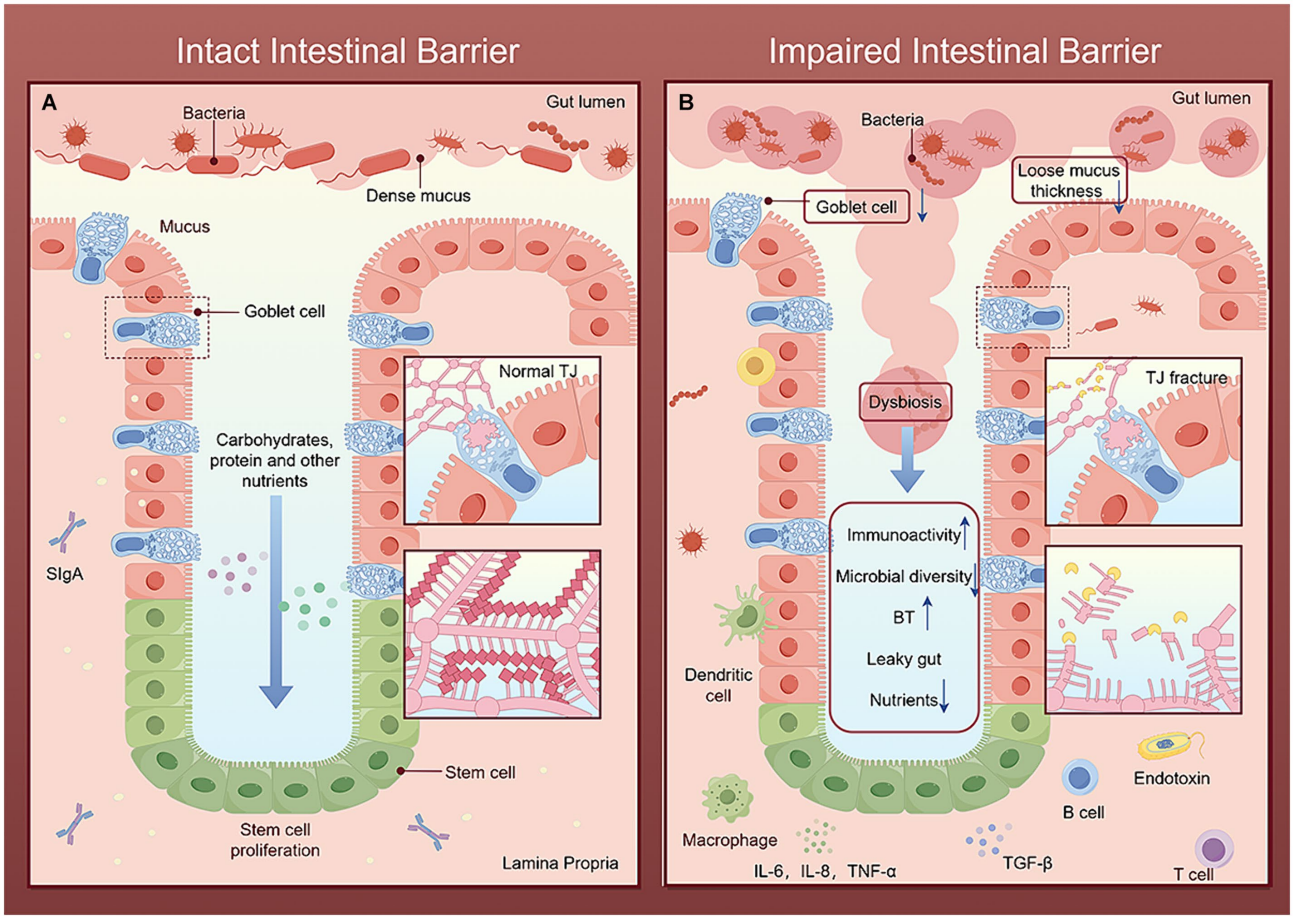
Comparison of intact and impaired intestinal barriers. The figure is drawn with Figdraw.com. **(A)** In the intact intestinal barrier, the intestinal microbial diversity is normal, the mucus layer limits the colonization and transfer of bacteria, the beneficial bacteria repel the invasion of pathogenic bacteria, and the complex structure of TJ together to maintain the selective permeability of intestinal epithelial cells, allowing nutrients and water ingested by the host to pass through and preventing harmful microorganisms and their metabolites from entering the portal vein through the intestinal barrier. Immune cells secrete various cytokines to timely remove invading pathogens and toxins and strengthen the intestinal barrier. **(B)** After the intestinal barrier is damaged, the mucus layer becomes loose and thick. Harmful microorganisms and their metabolites break through the mucus layer, destroy the intestinal tight-junction apparatus, and compete with beneficial bacteria and intestinal epithelial cells for nutrients, leading to increased intestinal permeability. Bacteria, toxins and other harmful molecules stimulate the intestinal immune barrier system, leading to enhanced immune activity and reduced microbial diversity. Finally, it further increases BT and intestinal leakage.

Potential harmful bacteria and their effector molecules, known as pathogen-associated molecular patterns (PAMPs), originating from the intestine, directly cross the damaged intestinal barrier and enter the liver, facilitated by various pattern recognition receptors. Activated liver cells release damage-associated molecular patterns, which, in conjunction with hepatic stellate cells (HSCs) and liver immune cells, particularly Kupffer cells, exacerbate liver inflammation and fibrosis (Guan et al., 2022). Therefore, the dysfunction of the intestinal barrier is an important prerequisite for PAMPs translocation to the liver and is a key factor influencing the pathogenesis of liver diseases. The intestinal barrier, composed of a mucous layer and an epithelial layer, together with the gut microbiota and immune system, maintains the normal function of the intestinal function (Tilg et al., 2022). These physical, immune, and biological barriers interact with each other (Figure 3), and any disruption at any crosslink will increase susceptibility to liver disease.

#### 3.2.1 Physical barriers

The mucus layer is mainly composed of secretion products from goblet cells, with its outer layer colonized by microbial communities and its inner layer containing minimal bacteria (Tsiaoussis et al., 2015). Mucus, predominantly composed of mucin (MUC), are divided into two types: transmembrane mucin (MUC1, MUC4, MUC15, MUC16, etc.) and secretory mucin (primarily MUC2) (Chopyk and Grakoui, 2020). Microorganisms competed for attachment sites and energy for colonization by degrading the O-and N-linked glycans and glycosaminoglycans of MUC2 (Martens et al., 2009). The protective effect of mucus in MUC2-deficient mice was significantly weakened, allowing direct entry of bacteria into epithelium and crypts, thereby inducing colitis-related cytokine expression (Johansson et al., 2008). Transmembrane mucin not only protected intestinal epithelial cells and acted as a lubricant but also provided immune protection and inhibits cell apoptosis (Constantinou et al., 2011; Paone and Cani, 2020). For example, MUC1 overexpression generated anti-MUC1 antibodies that could damage colon cells through cell-dependent cytotoxicity, leading to chronic intestinal inflammation such as ulcerative colitis (UC) (Apostolopoulos et al., 2015). Preventive treatment with anti-inflammatory milk fat globule membrane (MFGM) upregulated colonic MUC2, MUC4, Reg3g, and other gene expressions in mice, enhancing mucosal barrier integrity and reducing the risk of microbial-induced intestinal inflammation and secondary liver injury (Wu et al., 2022). Fecal samples from liver cirrhosis patients showed potential pathogenic bacteria rich in sialidase (e.g., *Veillonella atypica*, *Veillonella parvula*, and Streptococcus spp.) degrading MUC2 O-glycans, leading to barrier damage (Patel et al., 2022). Additionally, isoproterenol can reduce the number of goblet cells and mucus thickness in mice, impairing the intestinal mucosal epithelial and vascular barriers, facilitating BT to the liver and exacerbating liver damage (Sorribas et al., 2020). This multifaceted dysfunction of the intestinal barrier significantly contributes to the severity and complications of liver cirrhosis.

Intestinal epithelial cells include five specialized types: stem cells, goblet cells, neuroendocrine cells, Paneth cells, and intestinal absorptive cells (Pott and Hornef, 2012). They form a protein network that mediates selective permeability through transcellular and paracellular pathways (Tsukita et al., 2001). The protein network is composed of various adhesive complexes such as desmosomes, adhesive junctions (AJs), tight junctions (TJs), and gap junctions (Groschwitz and Hogan, 2009), among which TJ is recognized as the most important. Besides, Claudins, Occludin, and cytoskeleton-connected adhesive molecules (mainly microfilaments) considered as particularly important TJs (Tsiaoussis et al., 2015). Compared to healthy individuals, the expression levels of Occludin and Claudin-1 in intestinal epithelial cells were reduced in patients with liver cirrhosis, negatively correlated with the Child-Pugh score (Assimakopoulos et al., 2012). As a dynamic permeability tight barrier, intestinal epithelial TJs could prevent potential pathogenic bacteria, toxins, and other harmful substances from invading and permit the passage of nutrients, water, and ions (Hossain and Hirata, 2008). Mutation or defects in TJs cause TJPs contraction, enlarging intercellular spaces and enabling harmful macromolecules such as bacteria and toxins to infiltrate the portal vein, promoting the occurrence and development of liver cirrhosis (Twardowska et al., 2022). Additionally, oxidative stress is a key mechanism in intestinal barrier damage (Mohamed et al., 2021; Mo et al., 2022), inducing intestinal epithelial cell apoptosis, reducing the expression of TJPs, and increasing intestinal permeability (Li et al., 2022).

#### 3.2.2 Immune barriers

Following the disruption of the physical barrier structure, activation of the intestinal mucosal immune system occurs, with humoral immunity dominated by secreted immunoglobulin A (SIgA). SIgA not only prevents the recognition and uptake of bacteria, toxins, and other harmful molecules by intestinal mucosal epithelial cells (Mantis et al., 2011) but also contributes to the elimination of pathogens and toxins through intestinal mechanical peristalsis. Furthermore, it attenuates the systemic immune response and immune tolerance triggered by external antigenic stimulation and prevents the intestinal mucosal immune barrier system from eliciting an immune response to beneficial gut microbiota (Perez-Lopez et al., 2016). These intricate mechanisms involve T cells, interleukins (IL)-4, IL-6, transforming growth factor (TGF)-β, and tumor necrosis factor (TNF)-α within gut-associated lymphoid tissue (GALT) (Hooper and Macpherson, 2010). Prolonged pathogen stimulation of the immune system, coupled with inhibition of regulatory cells such as macrophages, dendritic cells, and M cells, disrupts immune homeostasis, thereby instigating intestinal inflammation. Multiple immune cells through complex interactions accelerates pro-inflammatory and fibrotic processes in the liver (Winer et al., 2016). Porowski et al. calculated the hepatic elimination ratio (LER) of IL-6, hepatocyte growth factor (HGF), TNF-α, and TGF-β introduced from the GALT and portal vein. In healthy individuals, LER values were 0.4, 0.2, 0.4, and 0.3 respectively, while in liver transplant patients, they were-0.1, −0.5, 0.1, and-0.2, respectively. Negative elimination rates indicate cytokine synthesis surpassing degradation in patients with liver cirrhosis (liver failure), with levels of these growth factors and cytokines closely related to the severity of liver cirrhosis (Porowski et al., 2015). Moreover, the severe immune deficiency induced by systemic inflammation and immune cell dysfunction in patients with liver cirrhosis plays a pivotal role in the pathogenesis of chronic acute liver failure (ACLF) (Martin-Mateos et al., 2019). In addition, endotoxins can severely damage the intestinal mucosal barrier and immune system of the body through oxidative stress (Wang and Wang, 2022). This particular subject was further elucidated in subsequent sections.

#### 3.2.3 Biological barriers

We explored the composition and changes in gut microbiota in the above discussion. Here, we delve into the interplay and mechanisms between the biological barrier, comprised of gut microbiota, and other barriers. Firstly, mucoglycans serve as nutrients for beneficial bacteria, enabling them to compete for mucosal colonization sites and inhibit pathogen colonization (Paone and Cani, 2020), thereby influencing mucus properties by interfering with glycosyltransferase expression, MUC2 glycosylation, and transmembrane mucin glycosylation (Schroeder, 2019; Pelaseyed and Hansson, 2020). Furthermore, microbial metabolites such as acetate and butyrate contribute to epithelial homeostasis by modulating goblet cells (Wrzosek et al., 2013). *Lactobacillus reuteri* mediates the PI3K/Akt Nrf-2/HO-1-NF-κβ and PKC-Nrf-2/HO-1-NF-κβ signaling pathways, enhancing the expression of TJPs, inhibiting TNF-induced apoptosis of intestinal epithelial cells (Zhou et al., 2022), and activating the Wnt/β-catenin pathway to stimulate the proliferation of intestinal epithelial cells, thereby regulating the physical barrier function of the intestine (Wu et al., 2020).

Microorganisms and the intestinal mucosal immune system interact with each other, jointly maintaining a symbiotic relationship between the host and microorganisms (Hooper et al., 2012). SIgA on the surface of the intestinal mucosa selectively binds to symbiotic bacteria, preventing their penetration of the epithelial barrier (Macpherson et al., 2000). Microbial colonization also facilitates the development and function of the intestinal immune system (O’Hara and Shanahan, 2006). The Toll-like receptor (TLR) family and nucleotide-binding oligomerization domain proteins Nod1 and Nod2 serve as primary receptor systems for microbial regulation of the innate immune system in the intestinal mucosa (Kelly and Conway, 2005). For example, LPS not only aids in the repair of damaged intestinal epithelium through MyD88-dependent processes (Rakoff-Nahoum and Medzhitov, 2007), but also activates the expression of the antimicrobial lectin Reg III γ, serving as an additional barrier to inhibit bacterial infiltration into the intestinal mucosa (Vaishnava et al., 2011). Therefore, excessive microbial stimulation may lead to the abnormal activation of intestinal immune cells, leading to intestinal inflammation. In summary, damages to the intestinal biological barrier inevitably affect the physical and immune barriers of the host, ultimately increasing the risk of various chronic liver diseases such as cirrhosis.

### 3.3 Abnormal metabolism of BAs

#### 3.3.1 Synthesis and pathways of BAs

BAs originate from cholesterol oxidation in the liver through two primary pathways. Under normal conditions, cholesterol 7α-hydroxylase (CYP7A1) initiates the classical pathway to produce the majority of primary BAs. CYP7A1 regulates the overall rate of BA synthesis, while sterol 12α-hydroxylase regulates the cholic acid (CA) to chenodeoxycholic acid ratio in the BA pool. In an adaptive response, alternative pathways, initiated by sterol-27 hydroxylase, play crucial roles in intestinal metabolism, cellular signaling, and microbial composition (Li and Chiang, 2014). Intestinal microbiota modify human BAs via four mechanisms: deconjugation of glycine or taurine, dehydroxylation, dehydrogenation, and epimerization (Guzior and Quinn, 2021), further activating BAs receptors. There is a close relationship between metabolic disorders of BAs and liver cirrhosis, along with its complications and intestinal dysbiosis.

#### 3.3.2 Bidirectional regulation of BAs and gut microbiota

Changes in gut microbiota composition and activity due to factors such as antibiotics, diet, and exercise significantly impact BA synthesis and signal transduction (Collins et al., 2023). Bacterial bile salt hydrolase (BSH), found in various gut bacteria like Clostridium, Enterococcus, Bifidobacterium, Lactobacillus, and Bacteroidetes, catalyzes the decoupling, oxidation, and 7α-dehydroxylation of conjugated BA, regulating the production level of secondary BAs (Ridlon et al., 2016). In addition, the composition and number of Firmicutes also affect secondary BA levels by altering 7α-dehydroxylation (Lee et al., 2022). Conversely, BAs influence microbiota composition and function, playing a decisive role in both quantitative and qualitative changes, as well as metabolic activities of gut microbiota. For example, studies suggest that glycine-and taurine-preferred BSH can inhibit the growth of *Clostridium difficile*, possibly by increasing the concentration of microbial conjugated BA, which inhibits the germination, growth, and toxin expression of *Clostridium difficile* (Foley et al., 2023). Moreover, BA administration in neonatal mice could significantly drive the maturation of the intestinal microbiota, improving its diversity and richness (Van Best et al., 2020). However, BAs may also harm intestinal bacteria due to their toxic effects, mediating oxidative stress and DNA damage (Collins et al., 2023). Overall, BA metabolism intricately links gut microbiota to physiology and pathology of the liver, offering a broader perspective for the treatment of liver diseases through the modulation of gut microbiota.

#### 3.3.3 Nuclear receptor FXR

FXR is a core regulatory factor in the biosynthesis of BAs and enterohepatic circulation. Dysfunction of the microbiota leads to decreased BA activity, reducing unconjugated BA and secondary BA production, weakening FXR activity, and exacerbating cirrhosis through various pathways: (1) In hepatocytes, FXR interacts with nuclear receptors such as peroxisome proliferator-activated receptors (PPAR)-α and liver X receptor (LXR)α to form heterodimers, regulating downstream target gene expression. Weakened FXR activity decreases the expression of PPAR-α and LXRα, negatively impacting liver energy metabolism (Preidis et al., 2017); (2) The inhibitory and regulatory effects on TLR4 transcription levels and NLRP3 inflammasome are weakened, promoting the production of inflammatory factors such as IL-1β and NOS2, further exacerbating the inflammatory response (Vavassori et al., 2009; Han et al., 2018); (3) Fat synthesis, high-density lipoprotein (HDL) formation, liver HDL uptake, and fatty acid β-oxidation, are affected, disrupting lipid metabolism and worsening liver steatosis and fibrosis (Ding et al., 2015); (4) Enhanced HSC activation increases collagen synthesis and deposition, accelerating liver fibrosis progression (Zhou et al., 2020). (5) Dysbiosis and the release of inflammatory markers (LPS) (Ridlon et al., 2015) further lead to BT, endotoxemia, and intestinal barrier damage, significantly correlating with liver cirrhosis progression and cognitive impairment (Zhang et al., 2022).

### 3.4 Microbial metabolites

The changes in gut microbiota observed during the occurrence and progression of liver cirrhosis are mainly attributed to the diversity, richness, and the presence of potential pathogenic byproducts such as endotoxins. Intestinal bacteria are the key source of LPS, and the liver is a vital site for LPS clearance. Therefore, abnormal production, kinetics and metabolism of LPS can reverse interfere with liver function and intestinal flora (Fuke et al., 2019). SCFA is the most abundant among all microbial metabolites, and about 500-600mmol of SCFAs are produced in the human intestine every day (Marrocco et al., 2022). SCFAs entering the liver supply more than 30% of the liver’s total energy source (Mann et al., 2024). As a tryptophan catabolite produced by the intestinal microbiota, indole is an indispensable signaling molecule regulating intestinal homeostasis (Roager and Licht, 2018). Although less than 1% of bacteria in the healthy gut contain genes for trimethylamine (TMA) synthesis, this is sufficient to regulate the balance of choline metabolism, indicating that these trace intestinal microorganisms and choline are essential for host health (Rath et al., 2017). Under anaerobic conditions, 1g of *Escherichia coli* produces 0.8 g of ethanol per hour, and excessive ethanol will significantly change the abundance of some intestinal flora, especially Bacteroidetes and Proteobacteria (Yan et al., 2023). In brief, metabolites produced by gut microbes are key mediators of gut microbiota-cirrhosis crosstalk.

**Figure 4:**
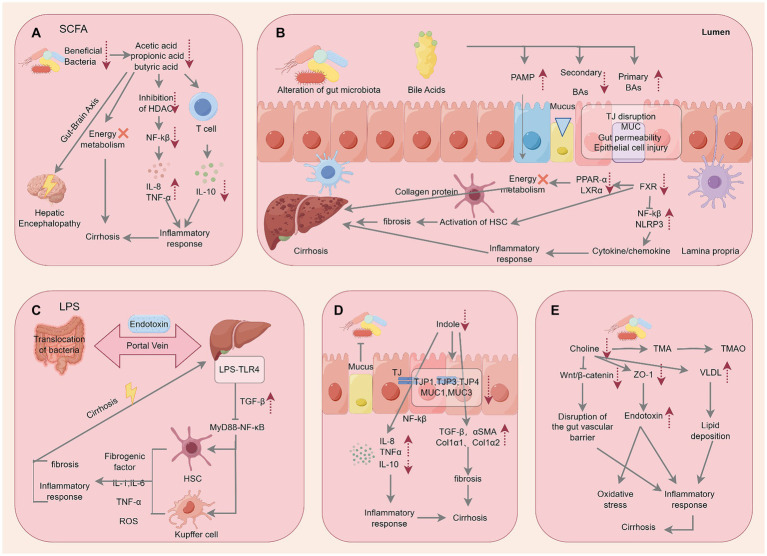
Specific mechanisms by which microbial metabolites act on cirrhosis. The figure is drawn with Figdraw.com.

The previously discussed physical, immune, and biological barriers within the intestine are critical in preventing harmful microorganisms and their metabolites from infiltrating the liver. However, dysbiosis or compromised intestinal barriers can lead to the abnormal accumulation of harmful metabolites in portal circulation, triggering a cascade of pro-inflammatory reactions associated with liver diseases (Ceccarelli et al., 2014). Yet, the specific mechanisms through which certain metabolites contribute to the occurrence and development of liver cirrhosis are not fully understood. Here, we delved into the pathological effects and mechanisms of SCFAs, endotoxins, indole, choline, and endogenous ethanol on the liver (Figure 4).

#### 3.4.1 Short chain fatty acids

The main products of anaerobic fermentation in the intestine are SCFAs, with acetic acid, propionic acid, and butyric acid being the most abundant (Vallianou et al., 2021). These SCFAs help maintain energy homeostasis through multiple pathways. Firstly, SCFAs, especially butyric acid, act as preferred energy sources for intestinal epithelial cells. Upon absorption, butyric acid is converted into cellular energy, inhibiting NLRP3 inflammasome activation and autophagy, thus protecting the intestinal barrier against LPS damage and maintaining the normal cell function (Feng et al., 2018). Moreover, acetate, propionate, and butyrate contribute to glucose, cholesterol, and lipid metabolism, directly impacting hepatic energy levels (Den Besten et al., 2013). Additionally, they indirectly influence the energy metabolism of peripheral organs by affecting the activity of hormones and the nervous system (Chambers et al., 2015; Perry et al., 2016). Therefore, SCFAs play an important role in human physiology and pathology (Koh et al., 2016).

SCFAs are closely related to the occurrence and severity of liver cirrhosis (Cao et al., 2023). There is a correlation between a decrease in the number of SCFA-producing bacteria compared with compensated patients and the occurrence of decompensated events (Bajaj et al., 2023). Reduced SCFA levels, particularly butyric acid, have been observed in fecal samples from cirrhotic patients, positively correlating with Child-Pugh classification (Jin et al., 2019). Furthermore, abnormal SCFA production correlates with liver cirrhosis severity. Diminished levels of beneficial bacteria such as Lachnospiraceae, Roseburia, Blautia, and Ruminococcaceae lead to decreased butyrate levels, impacting TJ and mucin (MUC) expression and altering intestinal barrier function and permeability. Consequently, pro-inflammatory cytokines were released into the liver (Woodhouse et al., 2018), affecting NF-κB signaling pathways and histone deacetylase inhibition (Neis et al., 2016). SCFAs have demonstrated an important role in regulating endothelial function, immune response, and vascular tone. The imbalance in SCFA production due to dysbiosis of the flora, particularly decreased Bacteroidetes abundance, disrupt these regulatory mechanisms, contributing to portal hypertension (Toshida et al., 2023).

#### 3.4.2 Endotoxin

Endotoxins, a component of LPS, constitute the outermost layer of Gram-negative bacteria cell walls, exerting diverse biological effects on ALD, viral hepatitis, cirrhosis, and its complications (Porras et al., 2017; Sehgal et al., 2020). Alcohol consumption and HFD-induced dysbiosis trigger potential pathogen translocation through the disrupted intestinal barriers. LPS binds to TLR4, stimulating Kupffer cells in the liver and the NF-κB pathway and promoting the expression of inflammatory factors, such as TNF-α, IL-1, and IL-6. On the other hand, LPS-TLR4 can increase the permeability of intestinal TJs and activate HSC, leading to inflammatory reactions in the intestine and liver. Besides, it also promotes the excessive secretion and deposition of collagen and other extracellular matrix proteins, ultimately leading to the development of liver cirrhosis (Roderburg and Luedde, 2014). Interestingly, LPS in activating immune signal transduction through TLR4-related pathways and affecting liver physiological functions is structurally dependent, indicating that a few LPS may act as antagonists to inhibit the occurrence of inflammatory responses (Di Lorenzo et al., 2019). In the gut microbiota of patients diagnosed with liver cirrhosis, increased abundance of the Enterobacteriaceae family, encoding heptyltransferase I (HpeI), a key enzyme involved in LPS synthesis, were observed and categorized into seven clusters. Specifically, the first cluster of HpeI serves as a biomarker for liver cirrhosis, offering diagnostic and treatment guidance from the perspective of gut microbiota (Lin et al., 2021).

Oxidative stress plays a crucial role in the progression of chronic liver disease to cirrhosis and its complications (Assimakopoulos et al., 2011). Liver damage weakens antioxidant effects, elevating oxidative stress and the production of reactive oxygen species (ROS), which subsequently harm the intestinal mucosa (Moustafa et al., 2009). It is specifically characterized by high levels of lipid peroxidation, protein oxidation, and an imbalance in glutathione redox status (Spahr et al., 2007). Damaged intestinal mucosa enhances permeability, promotes BT, coupled with collateral circulation and liver damage, finally leading to endotoxemia. The ensuing cycle exacerbates oxidative stress, intestinal barrier dysfunction, and organ damage (Liu et al., 2023), underscoring a significant correlation between liver cirrhosis, endotoxemia, and oxidative stress. Cheon et al. found that elevated endotoxin levels and subsequent increased ammonia levels activate the NLRP3 inflammasome through mitochondrial oxidative stress, resulting in neuroinflammation, brain edema, and the development of HE (Bajaj, 2014; Cheon et al., 2023). In addition, entero-derived endotoxins and oxidative stress are important factors in triggering portal hypertension in decompensated cirrhosis (Tripathi et al., 2018; Liu et al., 2023). Alterations in specific gut microbiota (Corynebacterium, Lautropia) and their excessive accumulation of metabolites, particularly LPS, are strongly associated with sepsis and related events in patients with decompensated cirrhosis (Philips et al., 2023). In addition, Sandler et al. proposed that LPS-induced inflammatory response and oxidative stress are predictive of progression to early compensated cirrhosis and end-stage liver failure in patients with chronic HBV or HCV infection (Sandler et al., 2011). Therefore, the exploration of drugs that target the regulation of LPS and its producing bacteria is expected to provide a therapeutic strategy for liver cirrhosis at different stages of progression.

#### 3.4.3 Indole

Indole can be produced by various gut microbiota such as *Escherichia coli*, Clostridium, and Bacteroidetes (Lee et al., 2015). Studies have found that indole can alter the composition of gut microbiota and increase gene expression of TJPs and mucins between epithelial cells, indicating enhanced intestinal epithelial barrier (Shimada et al., 2013). Moreover, indole can reduce TNF-α-mediated activation of NF-κβ, inhibit the expression of pro-inflammatory chemokine IL-8, and increase the expression of anti-inflammatory cytokine IL-10. Furthermore, it confers resistance to pathogen colonization, particularly against *Escherichia coli*, thereby attenuating intestinal inflammation (Bansal et al., 2010). Zhao et al. observed that indole intervention in HFD-treated rats resulted in significant downregulation of genes related to liver fibrosis and collagen synthesis (Zhao et al., 2019). However, subsequent studies have shown that indole or its metabolites do not consistently exhibit a beneficial role in liver disease. Liu et al. found that indole-3-propionic acid (IPA) could reduce the gap in gut microbiota diversity between CCl4-induced mice and healthy counterparts, activated HSCs and the TGF-β1/Smads signaling pathway, leading to hepatocyte apoptosis, increased expression of extracellular matrix components such as collagen I and α-SMA, ultimately promoting liver fibrosis (Liu et al., 2021). These contrasting findings underscore the necessity for thorough validation and consideration when evaluating the therapeutic potential of IPA in managing liver cirrhosis. Nevertheless, Li et al. analyzed 560 metabolites in serum metabolomics of patients with cirrhosis at different stages and found that IPA was the most significant indicator of decompensated metabolic biomarker reduction, suggesting that IPA can help accurately predict the stage of cirrhosis (Li et al., 2024).

#### 3.4.4 Trimethylamine

Gut microbiota metabolism involves the conversion of choline into TMA, which is absorbed by the host and subsequently converted into trimethylamine N-oxide (TMAO) in the liver (Arias et al., 2020). Imbalances in gut microbiota reduce the bioavailability of choline and increase portal venous influx of TMA, both implicated in metabolic abnormalities associated with liver cirrhosis (Albillos et al., 2020). Mourier et al. established a fatty liver model using a methionine/choline-deficient diet, revealing downregulation of the Wnt/β-catenin pathway in mice, leading to disruption of the intestinal vascular barrier (Mouries et al., 2019). Sawada et al. observed reduced expression of TJPs like ZO-1 in choline-deficient rats compared to controls, resulting in intestinal endotoxin accumulation in the portal vein, triggering liver inflammation and oxidative stress (Sawada et al., 2019). In addition, deficiencies in methionine and choline promote the accumulation of cholesterol and fat in the liver by inhibiting the assembly/export of very low-density lipoprotein (Simon et al., 2020). Excessive fat deposition in liver cells is the main cause of liver inflammation, implicating its role in diverse liver diseases (Li et al., 2023).

#### 3.4.5 Endogenous ethanol

In fact, Enterobacteriaceae and *Klebsiella pneumoniae* in the gut produce endogenous ethanol through the fermentation of carbohydrates, and ethanol metabolism produces toxic acetaldehyde (Salaspuro, 1997). Numerous studies have proved that when alcohol accumulates in the intestine due to long-term drinking, obesity and other factors, it will increase the expression of inflammatory cytokines to disrupt intestinal homeostasis, resulting in portal endotoxemia (Sheth et al., 2007; Kwon et al., 2014). In addition, ROS and ammonia produced by ethanol metabolism may cause oxidative stress and hepatocyte necrosis (Lieber, 1999). What’s more, its metabolite acetaldehyde may damage TJs of intestinal epithelial cells, destroy the intestinal barrier, cause pathological BT and intestinal leakage, and promote the progression of cirrhosis (Yan et al., 2023). In conclusion, endogenous ethanol can worsen liver inflammation through multiple pathways and has a significant effect on fibrosis.

## 4 Protective effects of TCM on liver cirrhosis induced by intestinal dysbiosis

Given the advantages of TCM, including its multi-component, multi-target, multi-path approaches, and mild effects, as well as its pharmacological activities like anti-inflammatory, antioxidant, anti-fibrotic, and hepatoprotective effects, it holds significant promise in the treatment of liver cirrhosis. Recent studies have highlighted the substantial potential of TCM in modulating ALD, NAFLD, liver cirrhosis, and liver cancer, primarily through targeted regulation of gut microbiota (Li et al., 2019; Liu et al., 2022). Currently, extensive research has been conducted on natural products and compounds for managing liver cirrhosis by targeting gut microbiota, with notable contributions from high-throughput screening and multi-omics research. Despite numerous studies, including clinical trials, demonstrating the significant therapeutic efficacy of TCM in treating liver diseases, the precise mechanism of action remains incompletely understood. This article was aimed to introduce the natural products (Table 2) and herbal compound formulations (Table 3) identified in recent research that focus on regulating gut microbiota to intervene in cases of liver cirrhosis.

**Table 2:** Summary of natural products targeting gut microbiota for the treatment of cirrhosis.

Type	Natural products	Research design	Regulatory strains	Possible mechanism	Reference
Polyphenolics	Resveratrol	CCl4 (0.5μL/g)-induced male mouse models were given RSV 30mg/kg daily for 4 weeks	Staphylococcus_lentus↓, Staphylococcus_xylosus↓	Enhance the expression of TJPs and intestinal barrier, reduce intestinal permeability and inhibit BT.	Li et al. (2022)
Alkaloids	Berbine		Firmicutes↑, especially *Clostridium scindens*↑	Maintain BA metabolic homeostasis, increase butyrate production to regulate lipid metabolism, relieve inflammatory response and immune disorders.	Liu et al. (2022)
	Palmatine	Based on its clinical dose design for humans (1.2g/60kg/ d), male rat models induced by CCl4 (1ml/kg) were given PAL, high dose (108mg/kg/ d) and low dose (54mg/kg/ d) respectively.	s_lactobacillus_reuteri↑, s_lactobacillus_murinus↑, s_lactobacillus_johnsonii↑	Enhance the expression of ZO-1, Claudin-1 and Occludin, decrease the expression of LPS, TNF-α, IL-6 and IL-1β, enhance intestinal barrier, and reduce inflammatory response, hepatocyte steatosis and collagen fiber deposition.	Qin et al. (2023)
Terpenoids	Curcumol	CCl4(5 mL/kg)-induced male mouse models were given daily intragastric administration of CU30 mL/kg (1 mg/mL CU solution and anhydrous ethanol) for 6 weeks	Bacteroidetes↓, Firmicutes↑, Proteobacteria↑	Inhibition of TLR4/NF-κβ signaling pathway and decrease the expression of downstream inflammatory factors.	Zheng et al. (2022)
	Ginkgolide A	CCl4(0.5ml/kg)-induced male Swiss albino mouse models were given GA100 mg/kg daily for 2 weeks	enterococcus↓, Bacteroidaceae↓	Inhibition of BT, restore the expression of PXR and related genes in the gut-liver axis, reduce the expression of TLR4/MyD88/NF-κβ axis and TNF-α, and improve the expression of ZO-1 、Occuldin	Mohandas and Vairappan (2020a, 2020b)
	Glaucocalyxin A	CCl4(0.01mL/g)-induced male C57BL/6J mice were intraperitoneally injected with GLA daily at a high dose (5mg/kg/ day) and a low dose (10mg/kg/ day) for 6 weeks	Bacteroidetes/Firmicutes ↑, Clostridia↓, Erysipelotrichales↓, Lachnospiraceae↓, Bacteroidia↑, Muribaculaceae↑	Reduce the expression of inflammatory factors and alleviate liver fibrosis.	Qu et al. (2023)
	EFT+SMP	CCl4(20%, 0.1ml/10g)-induced male mouse models were given EFT+SMP mixture 0.2ml/20g daily for 6 weeks	Firmicutes↓, Acfinobacteria↑, Candida phylum↑, Tenericutes↑	Increase intestinal microbial diversity in mice, reduce the production of bacterial metabolites LPS, LysoPE and LysoPC, inhibit kupffer cell activation and liver inflammatory response,then exerts potential anti-fibrotic effects.	Tan et al. (2020)
	Artesunate	CCl4、intragastric administration of 10% ethanol and HFD induced male rats were gavaged with AS (25 mg/kg) daily.	*Lactobacillus*↑, *Eubacterium* ↑, *Clostridium*↑, *Desulfotomaculum*↓, *Desulfosporosinus*↓	Increase intestinal microbial diversity, regulate intestinal permeability, reduce BT, reduce intestinal mucosal barrier damage and inflammatory response.	Chen et al. (2016)
	Cichorium pumilum Jacq Extract	5%2,4,6-trinitrobenzenesulfonic acid (60mg/kg) and 50% ethanol induced male rats were gavaged with CGEA at a high dose (150 mg/kg/ d) and a low dose (100mg/kg/d) for 2 weeks	Ochrobactrum ↓, Bacteroidetes↓, Ruminococcus ↑, *Acinetobacter* ↑, Bifidobacterium↑, Firmicutes↑, Proteobacteria↑, Acidobacteria↑	Inhibit the activation of MAPK-Akt signaling pathway, inhibit the phosphorylation of Akt and MAPKs proteins and the expression of inflammatory factors such as NO and IL-6 induced by LPS, reduce inflammation and anti-fibrosis.	Han et al. (2021)
Glycosides	Forsythiaside A	CCl4 (2mL/kg)-induced male C57BL/6 mouse model was administered with FTA (10 mL/kg) by gavage for 4 weeks	Firmicutes/Bacteroidetes↓, Mucispirillum↓, Lactobacillus↓, prevotellaceae_ UCG-001↑, Ruminococcus_1↑, Mucispirillum↑, Lactobacillus↑	Increase TJP expression and prevent intestinal injury, reduce endotoxin and inflammatory factor levels, maintain BA metabolic homeostasis, and increase SCFAs content (especially butyrate).	Fu et al. (2022)
Carbohydrates	Sodium alginate	CCl4(10%, 5mL/kg)-induced SPF male C57BL/6 mice were intraperitoneally injected with SA at high dose (100 mg/kg) and low dose (50 mg/kg) for 4 weeks	Lactobacillus↑, Lachnospiraceae↑, Faecalibaculum↑, Marvinbryantia↑, Phascolarctobacterium↑, Oscillospiraceae↑, Monoglobus↓, Erysipelotrichaceae↓, Ileibacterium↓	Regulate intestinal flora, protect intestinal barrier, reduce inflammation level, reduce liver fibrosis and liver injury.	He et al. (2023)
	*Lycium barbarum* L. oligosaccharides	CCl4(10mL/kg)-induced male C6BL/2019J mouse were gavaged with LBO dissolved in normal saline (200mg/kg bw)	bacillus ↑, Tyzzerella↑, Fournierella↑, corobacteriaceae UCG-002↑, but could not fully restore the intestinal flora of mice to a healthy state	Enhance intestinal barrier function, reduce intestinal permeability and inflammatory response, and significantly alleviate liver fibrosis and mitochondrial metabolism disorders	Zhang et al. (2023)	
	Dendrobium officinale polysaccharide	CCl4 (30%, 2mL/kg)-induced male rats were treated by gavage of DOP solution at high (800 mg/kg), middle (400 mg/kg) and low (200 mg/kg) dose twice a week for 8 weeks		The expression of Claudin-1, ZO-1, Bcl-2 and other TJP proteins was up-regulated, and the expression of Bax and caspase-3 proteins was down-regulated, which may benign regulate the intestinal mucosal barrier. At the same time, inhibition of LPS-TLR4-NF-κB signaling pathway, decreased the expression of TGF-β and TNF-α, increased the expression of IL-10, and decreased the expression of α-SMA and collagen I.	Wang et al. (2020)
	Garlic polysaccharide	ALF mouse model was established by gavage of alcohol (56%, 6 mL/kg) for 30 days and then treated with GP, high dose group (250 mg/kg), low dose group (150 mg/kg).	Lachnospiraceae↑, Lactobacillus↑, Facklamia↓, Firmicutes↓	Remodeled intestinal microecology, down-regulated the expression of TGF-β1 and TNF-α proteins, increased the content of decorin, inhibited the activation of HSCs, and reduced the production of ECM.	Wang et al. (2018)

CCl4, carbon tetrachloride; TJ, tight junction; BT, bacterial translocation; TLR, toll-like receptor; NF-κβ, nuclear factor kappa-betta; PAL, palmatine; ZO, zonula occludens; LPS, lipopolysaccharide; LysoPE, lysophosphatidylethanolamine; LysoPC, lysophosphatidylcholine;TNF, tumor necrosis factor; IL, interleukin; GA, ginkgolide A; PXR, Pregnane X receptor; MyD88, myeloid differentiation factor-88; GLA, glaucocalyxin A; EFT, *E. fukienensis* Hsu.; SMP, *S. miltiorrhiza* Bge; AS, artesunate; DOP, *Dendrobium officinale* polysaccharide; GP, garlic polysaccharide; CGEA, *Cichorium pumilum* jacq; MAPK, mitogen-activated protein kinases; FTA, forsythiaside A; SCFA, short-chain fatty acid; BA, bile acid; LBO, *Ycium barbarum L. oligosaccharides*.

**Table 3:** Summary of Herbal compound prescriptions targeting gut microbiota for the treatment of cirrhosis.

Herbal compound prescriptions	Composition	Regulatory strains	Possible mechanism	Reference
BJJP	turtle shell, donkey-hide gelatin, nidus vespae, pillbug, ground beetle, dung beetle, saltpeter, bupleurum, scutellaria, pinellia, codonopsis, rhizoma zingiberis, magnolia officinalis, cassia twig, radix paeoniae alba, rhizoma belamcandae, peach kernel, cortex moutan, *rheum officinale*, trumpet creeper, semen lepidii, pyrrosia leaf, fringed pink	Bifidobacteria↑, Lactobacillus↑, Faecalibacterium↑, Blautia↑, *Escherichia coli*↓, Bacteroides↓, Ruminococcus↓, Parabacteroides↓, Prevotella↓	Increase carbohydrate and vitamin metabolite levels in mice, reduce intestinal permeability and inflammatory response, improve mitochondrial dysfunction and liver fibrosis	Chi et al. (2023)
DHZCP	rhubarb, soil locust, leech, locust, grub, peach kernel, true lacquertree dried lacquer, scutellaria baicalensis, radix paeoniae alba, amygdala amara, rehmannia, licorice	Firmicutes/Bacteroidetes↓,Prevotella↑,alloprevotella↑,phascolarctobacterium↑,muribaculaceae unclassified↑,lachnospiraceae unclassified↑,clostridiales unclassified↑,desulfovibrio↓, colidextribacter↓	Regulate microbial metabolites, enhance the damaged intestinal barrier, reduce BT, and reduce inflammatory response and liver fibrosis by inhibiting the TLR4/MyD88/NF-κβ pathway	He et al. (2024)
YCWLP	artemisia Capillaris Herba, Polyporus Umbellatus, Alismatis Rhizoma, Atractylodes Macrocephalae Rhizoma stir-fried with wheat bran, Poria, Cinnamomi Ramulus	Christensenella↑, Ruminococcus↑, Barnesiella↑, Bifidobacterium↑, Coprococcus↑, Anaerostipes↑,	Increase the production of butyrate, improve the metabolism of amino acid, sphingolipid and glucose in rats by regulating related microbial metabolites, and then reduce the content of ammonia in the body, regulate inflammation and immune response	Zhang et al. (2021a, 2021b)
GSG	Codonopsis radix, Radix buuri, Radix sinensis, Radix *salvia miltiorrhiza*, Atractylodes atractylodes, Polygonum cuspetatum, peach kernel, Fructus aurantii, Poria, dandelion, Prunella sinensis, Biejia, Radix paeoniae alba	Bacteroidetes/Firmicutes↑, Lactobacillus↑, Akkermansia↑, Bacteroides↑, Oscillospira↑, Acinetobacter↑, Allobaculum↓	Reduce intestinal permeability, reduce BT, and enhance the intestinal barrier	Zhao et al. (2021)
ZGHYD	Astragalus membranaceus, Turtle shell, Three Lengs, Zedoary turmeric, Chuanxiong, *Salvia miltiorrhiza*, Hedyotis diffusa, Scutellaria barbata	Eubacteriaceae↑, Lactococcus↑, Micrococcaceae↑, Rothia↑, Eubacteriaceae↑, Rothia↑, Acidaminococcus↑, Fusobacteriaceae↓, erysipelotricaceae↓	Promote SCFAs production, enhance intestinal barrier, inhibit inflammatory response, and alleviate liver injury and fibrosis	Wenlin et al. (2023)
QJ	Astragali radix, Angelicae sinensis radix, Trionycis carapax, Eupolyphaga steleophaga, Salviae miltiorrhizae radix et rhizoma, Carthami flos, Persicae semen, Sparganii rhizome, Curcumae rhizome, Glycyrrhizae radix et rhizome	Lactobacillaceae↑, rumen bacterium↑, Rikenellaceae↑, Limosilactobacillus Reuteri↑,*Escherichia coli*↓, Muribaculaceae↓, Prevotellaceae↓	Restore the structure of intestinal tissue, increase the expression levels of Claudin-1, Occludin and ZO-1, restore the TJ apparatus damaged by CCl4, and inhibit liver inflammation and liver fibrosis by repairing the intestinal epithelial barrier	Li et al. (2024)

BJJP, Biejiajian Pill; DHZCP, Dahuang Zhechong Pill; YCWLP, YinChenwuling Powder; GSG, Ganshuang granules; ZGHYD, Zhenggan Huayu Decoction; QJ, Qijia Rougan decoction; BT,bacterial translocation; TLR, toll-like receptor; MyD88, myeloid differentiation factor-88; NF-κβ, nuclear factor kappa-betta; SCFA, short-chain fatty acid.

### 4.1 Natural products of TCM

#### 4.1.1 Polyphenols

Resveratrol (RSV) is primarily derived from natural plants such as *Polygonum cuspidatum*, Cassia seed, and grapes (Zhang et al., 2021). RSV has various pharmacological activities, including anti-inflammatory, antioxidant, anti-lipogenic, and anti-tumor effects, which could be closely related to its capacity for modulating gut microbiota, thereby ameliorating liver cirrhosis (Kim et al., 2011; Gambini et al., 2015). Recently, Li et al. found that in a CCl4-induced liver fibrosis rat model, RSV reversed the reduced expression of ZO-1, Occludin, and other proteins and mRNA induced by CCl4, accelerating the repair of damaged intestinal mucosal barriers. Moreover, while *Staphylococcus xylosus* and *Staphylococcus lentus* accelerate BT and enhance intestinal permeability, RSV inhibits the excessive proliferation of *Staphylococcus lentus* by inhibiting its biofilm formation, leading to the regulation of intestinal flora imbalance, adjustment of gut microbiota balance, and substantial amelioration of liver fibrosis (Li et al., 2022).

#### 4.1.2 Alkaloids

Berberine (BBR) a quaternary ammonium alkaloid derived from Rhizoma coptidis, has become a novel therapeutic approach in recent years (Song et al., 2020). It relies on its antibacterial, anti-inflammatory, anti-fibrotic, and anti-hyperlipidemic activities to target the regulation of gut microbiota in the treatment of liver diseases. BBR can enhance intestinal anaerobic bacteria to stimulate butyrate production, elevate the levels of beneficial bacteria (especially Clostridium) in the intestines, thereby reducing host liver inflammation, regulating lipid metabolism, and improving liver fibrosis. Additionally, BBR serves as an FXR and TGR5 agonist, pivotal in modulating the metabolic homeostasis of BAs, thereby intervening in liver cirrhosis by mediating the crosstalk between BAs metabolism and gut microbiota (Liu et al., 2022). However, studies have indicated that BBR itself can induce dysbiosis of the microbiota, leading to mild diarrhea in the host (Yue et al., 2019). Therefore, in future, special attention should be paid to the adverse reactions or intolerance of BBR in the drug development.

Corydalis saxicola Bunting (CSB) has significant therapeutic benefits in various liver diseases (Qin et al., 2023). Recently, Qin et al. demonstrated for the first time that palmitine (PAL) is the main active component of CSB and plays a pivotal role, particularly in conferring anti-fibrotic attributes. Further analysis using metagenomic sequencing methods revealed that the anti-fibrotic properties of PAL depended on the gut microbiota and could significantly reverse the decrease in the abundance of s_*Lactobacillus_reuteri*, s_*Lactobacillus_murinus* and s_*Lactobacillus_johnsonii* induced by CCL4. These three bacteria were directly negatively correlated with the degree of liver fibrosis. In addition, the levels of LPS, TNF-α, IL-6 and IL-1β in the liver of mice were reduced to varying degrees after PAL treatment, repair the intestinal barrier, and increase the immune response of intestinal epithelial cells against inflammatory stimuli, thereby elucidating the specific mechanism underlying PAL’s hepatoprotective effects (Qin et al., 2023).

#### 4.1.3 Terpenoids

Curcumol (CU), a sesquiterpenoid compound extracted from turmeric, has garnered attention for its crucial anti-inflammatory, anti-fibrotic, and immunomodulatory roles in liver and intestinal diseases (Liu et al., 2019; Yang et al., 2020). Recent CU can effectively alleviate liver cirrhosis (Zhang et al., 2024), alter the fecal microbiota composition in rats afflicted with liver fibrosis, reduce the abundance of Bacteroidetes, increase the abundance of Firmicutes and Proteobacteria, and inhibit the activity of TLR4/NF-κβ signaling pathway, leading to reduced expression of inflammatory factors (IL-6, TNF-α, IL-8) (Zheng et al., 2022). These findings suggest that CU has a potential as an alternative therapeutic drug targeting the liver and intestine.

Ginkgolide A (GA), a diterpenoid compound extracted from the roots and leaves of *Ginkgo biloba* trees, has been widely used in clinical practice to treat cardiovascular and neurological disorders (Wang et al., 2023). However, recent studies have found that GA has great potential in managing liver diseases (Ye et al., 2016; Jeong et al., 2017). Enterococcus and Bacteroides, but not Lactobacillus, were involved in GA regulation of intestinal flora imbalance in cirrhotic mice. It can also activate the progesterone X receptor (PXR), a potential therapeutic target for liver cirrhosis, thereby upregulating the expression of PXR in the liver and intestines. By improving the expression of TJPs such as ZO-1 and Occludin, inhibiting pathological translocation of intestinal bacteria, and regulating the inflammatory cytokine environment within the liver, GA plays an important role in maintaining the balance of the gut-liver axis during the treatment of liver cirrhosis (Mohandas and Vairappan, 2020a,b).

Glaucocalyxin A (GLA), a natural n-pentane diterpene isolated from the aerial parts of Rabdosia japonica (Chen et al., 2021), has recently been implicated in improve gut microbiota dysbiosis in a mouse model of liver fibrosis. GLA treatment could decrease the Bacteroidetes to Firmicutes ratio. At the class level, it decreased abundance of Clostridia and increased availability of Bacteroidia. At the order level, the abundance of Erysipelotrichales decreased, while that of Bacteroidia increased. At the family level, Lachnospiraceae became less abundant in the GLA group, whereas the presence of Muribaculaceae and Bacteroidaceae increased. GLA can also reduce the levels of inflammatory factors that are closely related to the gut microbiota, such as TNF-α, TGF-β, and ROS, thereby reducing the extent of liver fibrosis and addressing the imbalance of intestinal flora caused by liver diseases (Qu et al., 2023).

In addition, Yang et al. explored the synergistic effect of a blend of triterpenoids extracted from E. fukienensis Hsu. (EFT) and phenolic acids derived from the roots of *S. miltiorrhiza* Bge (SMP) on liver fibrosis. Their findings underscored a significant reduction in the Firmicutes/Bacteroidetes ratio in the EFT+SMP group compared to the model group, concomitant with a decrease in the levels of gut microbiota metabolites lysophosphatidylethanolamine (LysoPE) and lysophosphatidylcholine (LysoPC), both of which have pro-fibrotic effects, thereby inhibiting the activation of Kupffer cells. These findings suggest that the potential association between EFT+SMP and gut microbiota may serve as a mechanism for the management of liver fibrosis (Tan et al., 2020).

Artesunate (AS) is synthesized based on dihydroartemisinin, a derivative of artemisinin, and is mainly used for malaria treatment (Rueangweerayut et al., 2012). AS has beneficial therapeutic effects on liver diseases (Kong et al., 2019). Chen et al. found that AS intervention group exhibited a significant increase in intestinal microbial diversity (primarily *Lactobacillus*), compared to the cirrhosis model group, and resulted in a deceased incidence and quantity of BT, contributing to the protection of the intestinal barrier, reduction of inflammation, and mitigation of liver injury. These findings demonstrated that AS effectively reduced the prevalence of liver cirrhosis induced by variouspathogenic factors, such as CCl4, alcohol, and HFDs (Chen et al., 2016).

Previous studies have underscored the significant anti-liver fibrosis activity and “probiotic-like effect” of the ethyl acetate extract of *Cichorium pumilum* Jacq (CGEA) (Qin et al., 2014; Lee et al., 2015). Therefore, building upon the gut-liver axis concept, Chang et al. further explored the effects of CGEA on the gut microbiota and intestinal barrier and discovered that CGEA promoted the growth of *Bifidobacteria*, increased the abundance of *Ruminococcus*, and consequently improved the homeostasis of the gut-liver axis. It is essential to highlight that lactate, the main compound in CGEA, is significant for the anti-inflammatory process. By inhibiting the phosphorylation of mitogen-activated protein kinase (MAPK) and Akt signaling pathways triggered by LPS, CGEA can effectively suppress the expression of inflammatory factors such as IL-6 and NO, thereby alleviating the liver’s inflammatory response (Han et al., 2021).

#### 4.1.4 Carbohydrates

Sodium alginate (SA), a natural polysaccharide extracted from seaweed or Sargassum of brown algae post-iodine and mannitol extraction, has emerged as a potential drug for liver protection and regeneration promotion (Li et al., 2022; Xia et al., 2023). Chen et al. underscored that liver fibrosis resulted in reduced gut microbiota diversity in rats, elevated colonization of harmful bacteria (such as Erysipelotrichaceae, Helicobacter, and Monolobus), and compromised the intestinal barrier. SA intervention remedied these pathological changes and increase the abundance of beneficial bacteria (such as Lactobacillus, Lachnospiraceae, and Eubacteria), thus improving liver fibrosis and inflammatory responses by restoring the stability of gut microbiota (He et al., 2023).

*Lycium barbarum* L. oligosaccharides (LBO) are oligosaccharides derived from *Lycium barbarum*. CCl4-induced liver fibrosis in mice results in increased ROS expression and mitochondrial dysfunction (Bi et al., 2024). Zhang et al. conducted further research and discovered a potential association between the pathological changes observed in liver fibrosis and dysbiosis of the gut microbiota. LBO could effectively treat liver fibrosis, mainly due to the restoration of gut microbiota, significantly increasing the enrichment of commensal bacteria Bacillus, Tyzzerella, Fournierella and Coriobacteriaceae UCG-002, further improves metabolic disorders caused by bacterial dysbiosis, repairs intestinal mucosal damage, regulates colonic epithelial permeability, and enhances mitochondrial function (Zhang et al., 2023).

Dendrobium officinale polysaccharide (DOP) have been previously shown to have anti-inflammatory, anti-oxidative, immune, hypoglycemic and other pharmacological properties (Liu et al., 2021; Wang et al., 2022; Chen et al., 2023), and play a significant therapeutic effect on acetaminophen induced liver injury by targeting the intestinal mucosal barrier (Lin et al., 2018). Subsequently, Wang et al. further explored and found that the mechanism of DOP in maintaining intestinal balance involved regulating the expression levels of TJPs such as occludin, claudin-1, ZO-1 and caspase-3 proteins, inhibiting the activation of LPS-TLR4-NF-κB signaling pathway, and reducing the content of inflammatory factors TGF-β and TNF-α, increasing the expression of anti-inflammatory factor IL-10, which significantly reduces the expression of α-SMA and collagen I, thereby reducing hepatocyte apoptosis and liver fibrosis (Wang et al., 2020).

Garlic, which is commonly used as a food spice, is also one of the traditional Chinese medicines and can be widely used in the treatment of cancer, nervous system and various metabolic diseases (Zhang et al., 2020; Tedeschi et al., 2022). In an experimental study on alcoholic liver fibrosis (ALF), garlic polysaccharide (GP) reversed the negative effects of changes in intestinal dominant flora on ALF, enriched Lachnospiraceae and Lactobacillus again, and reduced the diversity of potential pathogenic bacteria (Firmicute and Facklamia). It also regulates the protein expression of TGF-β1 (a key pro-fibrotic factor), TNF-α, and decorin (an anti-fibrotic agent), inhibits HSCs activation and reduces extracellular matrix (ECM) accumulation, ultimately demonstrating its hepatoprotective activity (Wang et al., 2018).

#### 4.1.5 Glycosides

FTA is a glycoside compound extracted from the dried fruit of *Forsythia suspensa* and serves as a natural hepatoprotective agent (Gong et al., 2023). Fu et al. found that FTA not only restored the disrupted structure of intestinal microbiota, specifically, Ace index increased significantly, Shannon index decreased significantly, Lachnospiraceae_NK4A136_group, Ruminococcaceae_UCG-014, Lactobacillus、Bacteroides, Prevotellaceae_UCG-001, Helicobacter and Alloprevotellabut were adjusted to benefit the health of the host, also promoted the production of SCFA (particularly butyric acid). Furthermore, FTA reduced the levels of inflammatory factors such as LPS and TNF-α in serum, upregulated the mRNA expression of ZO-1, Claudin-1, and Occludin in a dose-dependent manner, thereby mitigating intestinal permeability. Meanwhile, FTA regulates the expression of genes related to BAs metabolism (such as FXR, CYP7A1.), thereby facilitating the communication between gut microbiota and the liver, underscoring its hepatoprotective and anti-fibrotic properties (Fu et al., 2022).

### 4.2 Herbal compound prescriptions

Limited research on the regulation of intestinal microbiota by TCM compounds for liver cirrhosis has been conducted. Biejiajian pill (BJJP), composed of 23 Chinese herbs (including turtle shell, donkey-hide gelatin, nidus vespae, pillbug, ground beetle, dung beetle, saltpeter, bupleurum, scutellaria, pinellia, codonopsis, rhizoma zingiberis, magnolia officinalis, cassia twig, radix paeoniae alba, rhizoma belamcandae, peach kernel, cortex moutan, *rheum officinale*, trumpet creeper, semen lepidii, pyrrosia leaf, fringed pink)., showed promising results in a prospective randomized controlled trial. After 48 weeks of BJJP treatment, patients with hepatitis B cirrhosis/liver fibrosis showed a significantly higher improvement rate of fibrosis compared to the model group. This improvement was associated with an increase in the abundance of beneficial bacteria (such as Bifidobacterium, Lactobacillus, and Faecalibacterium) and a decrease in the abundance of potential pathogenic bacteria (such as *Escherichia coli*, Bacteroidetes, Ruminococcus, and Parabacteroides), demonstrating the close relationship between microbial community composition, reduced inflammatory response, and improved liver fibrosis and injury (Chi et al., 2023).

Moreover, He et al. investigated Dahuang Zhechong pill (DHZCP, consisting of rhubarb, soil locust, leech, locust, grub, peach kernel, true lacquertree dried lacquer, scutellaria baicalensis, radix paeoniae alba, amygdala amara, rehmannia, and licorice), and found that it could maintain the normal Firmicutes and Bacteroidetes ratio in liver fibrosis rats. DHZCP increased the abundance of beneficial bacteria, especially those producing SCFA (such as Prevotella, Ruminococcus, and Clostridium), while reducing the abundance of harmful bacteria (Desulfovibrio, Colidextribacter) and the production of LPS. DHZCP also regulated the protein and mRNA expression levels of ZO-1, TLR4, and MyD88, significantly improved the intestinal barrier, and reduced BT and endotoxemia, underscoring the anti-liver fibrotic effects of DHZCP on gut microbiota (He et al., 2024).

YinChenWuling Powder (YCWLP) is a combination of artemisia Capillaris Herba, Polyporus Umbellatus, Alismatis Rhizoma, Atractylodes Macrocephalae Rhizoma stir-fried with wheat bran, Poria, and Cinnamomi Ramulus. It is worth noting that hippuric acid can serve as a biomarker of host metabolic health, and its production level is strongly correlated with gut microbiota activity (Wang et al., 2020). Zhang et al. suggested a decrease in hippuric acid levels in rats with CCl4-induced liver fibrosis and a significant increase after the treatment with YCWLP (Tedeschi et al., 2022). YCWLP could alleviate the severity of CCl4-induced liver fibrosis by reshaping the gut microbiotaby regulating bacterial genera (such as *Christensenella*, *Ruminococcus*, and *Barnesiella*) associated with liver fibrosis. Moreover, by increasing the production level of butyrate, YCWLP could regulate the microbial metabolites in rat feces. This, in turn, helped correct metabolic disorders of amino acids, sphingolipids, and glucose, thereby reducing the generation and accumulation of ammonia. These effects highlight the potential of YCWLP in maintaining its hepatoprotective, anti-inflammatory, and immunomodulatory effects (Zhang et al., 2021a).

Ganshuang granules (GSG), composed of Codonopsis radix, Radix buuri, Radix sinensis, Radix *salvia miltiorrhiza*, Atractylodes adactylies, Polygonum cuspetatum, peach kernel, Fructus aurantii, Poria, dandelion, Prunella sinensis, Biejia, and Radix paeoniae alba, have good therapeutic effects on liver fibrosis and cirrhosis (Brial et al., 2021; Zhang et al., 2021b). Zhao et al. further investigated its underlying mechanism by targeting intestinal flora and found that GSG could mitigate xidative stress, inflammatory response, and liver fibrosis in a CCl4-induced rat model. The potential mechanisms in treating liver fibrosis may be closely related to reshaping the homeostasis of gut microbiota and enhancing the intestinal barrier (Zhao et al., 2021).

Zhenggan Huayu decoction (ZGHYD) is a herbal formulation composed of *Astragalus membranaceus*, Turtle shell, Three Lengs, Zedoary turmeric, Chuanxiong, *Salvia miltiorrhiza*, *Hedyotis diffusa*, and *Scutellaria barbata*. Research has shown that ZGHYD has the potential to increase the abundance of beneficial bacteria, while it may also enrich certain pathogenic bacteria, specifically *Enterococcus*, *Rothia*, and *Leuconostoc*. *Rothia*, interestingly, was positively correlated with the levels of type III procollagen peptide (PIIINP) and spleen thickness. PIIINP levels and spleen thickness, which are indicators of liver damage, showed a positive correlation with the presence of these pathogens. Enterococcus is a gram-negative bacterium whose abundance is positively correlated with LPS, serum IL-6, IL-1, and other inflammatory cytokines. These findings suggest that ZGHYD may not completely correct the dysregulated intestinal flora, but adjust the composition of certain pathogenic and beneficial bacteria. Nevertheless, ZGHYD demonstrated a notable positive effect in reversing liver fibrosis, surpassing the efficacy of Entecavir as a standalone treatment (Wenlin et al., 2023).

Recent studies have confirmed that Qijia Rougan decoction, composed of Astragali radix, Angelicae sinensis radix, Trionycis carapax, Eupolyphaga steleophaga, Salviae miltiorrhizae radix et rhizoma, Carthami flos, Persicae semen, Sparganii rhizome, Curcumae rhizome, and Glycyrrhizae radix et rhizome, has the effect of alleviating liver inflammation and anti-fibrosis. It was further found that the mechanism involved in targeting 10 species of bacteria and 37 metabolites in the intestinal tract (the most critical bacteria were Turicibacter, Faecalibaculum, Prevotellaceae UCG 001 and Peptococcaceae). After QJ, the intestinal tissue structure of mice returned to normal, the expression levels of Claudin-1, Occludin and ZO-1 increased, the TJ apparatus damaged by CCl4 was restored, and liver inflammation and liver fibrosis were inhibited by repairing the intestinal epithelial barrier (Li et al., 2024). Targeting enterohepatic circulation has become a potential drug for the treatment of liver fibrosis/cirrhosis.

### 4.3 Strengths and limitations

A growing body of research underscores the significant therapeutic impact of TCM on gut microbiota and liver cirrhosis, driving ongoing exploration into new Chinese herbal remedies. However, numerous herbal medicines face challenges in clinical application due to their limited bioavailability. It has been observed that a favorable gut microenvironment can significantly influence the metabolism of active ingredients in TCM, thereby enhancing their bioavailability (Gong et al., 2020).

Despite the rapid expansion of research on TCM interventions targeting gut microbiota in liver cirrhosis, we still face many problems that need to be solved in the future. Firstly, there is a scarcity of clinical studies investigating the effects of TCM on gut microbiota, with most studies conducted in animal models or *in vitro* settings. Moreover, it is possible that other herbal extracts may possess similar bioactivity in the treatment of liver fibrosis, and these potential effects may still be undiscovered. Consequently, the next step should involve conducting extensive clinical research to explore the therapeutic potential of alternative medicines through clinical research. Secondly, the quality of existing literature varies, and not all TCM treatments have demonstrated beneficial effects on gut microbiota and liver health, as evidenced by the example of ZGHYD discussed earlier. Therefore, it is necessary to further conduct high-quality research to explore the efficacy, safe dosage, and adverse effects of TCM on the human body.

## 5 Conclusion

In conclusion, regardless of physiological or pathological conditions, there exists a close relationship between gut microbiota and liver cirrhosis. It is noteworthy that patients with liver cirrhosis experience significant pathological changes in the abundance of gut microbiota, intestinal barrier function, BAs metabolism, and microbial metabolites. In addition, there are varying degrees of liver and systemic inflammatory responses, liver fibrosis, and metabolic dysregulation. However, regulating the imbalanced gut microbiota has shown promising effects in reversing these pathological changes observed in both the gut and liver associated with cirrhosis. Thus, the gut microbiota emerges as a pivotal factor in liver cirrhosis.

Recent studies have shed light on the efficacy of TCM in targeting gut microbiota to treat liver cirrhosis. This review summarized the specific mechanisms involved in modulating gut microbiota for the treatment of liver cirrhosis using TCM. Various compounds, including polyphenols, alkaloids, terpenoids, and glycosides, have been investigated. These TCM components impact liver cirrhosis through four primary mechanisms: (1) regulating gut microbiota composition and abundance; (2) Enhancing the intestinal barrier, reducing intestinal permeability, and safeguarding against the entry of PAMPs into the portal circulation; (3) modulating genes involved in BA metabolism and activating the FXR receptor to maintain the metabolic homeostasis of BAs; (4) influencing the production and expression of key metabolites (such as LPS and SCFA) by modulating microbe-derived metabolites, thereby mitigating damage through the portal system.

While research in this field is still in its early stages, the therapeutic potential of TCM targeting gut microbiota modulation in liver cirrhosis is undeniable. Given its multifaceted composition and ability to address multiple factors, TCM has the capacity to slow down the progression of liver cirrhosis and inspire novel ideas in drug development. Thus, it represents a significant milestone in gut microbiota-based liver cirrhosis research.

## Author contributions

SS: Writing – original draft, Writing – review & editing. GZ: Writing – original draft. SL: Writing – review & editing. JS: Writing – original draft, Writing – review & editing.
